# Depression, anxiety and post-traumatic stress during the 2022 Russo-Ukrainian war, a comparison between populations in Poland, Ukraine, and Taiwan

**DOI:** 10.1038/s41598-023-28729-3

**Published:** 2023-03-03

**Authors:** Agata Chudzicka-Czupała, Nadiya Hapon, Soon-Kiat Chiang, Marta Żywiołek-Szeja, Liudmyla Karamushka, Charlotte T. Lee, Damian Grabowski, Mateusz Paliga, Joshua D. Rosenblat, Roger Ho, Roger S. McIntyre, Yi-Lung Chen

**Affiliations:** 1 Faculty of Psychology, SWPS University of Social Sciences and Humanities, Katowice, Poland; 2 Department of Psychology, Ivan Franko National University of Lviv, Lviv, Ukraine; 3 Department of Psychological Medicine, Yong Loo Lin School of Medicine, National University of Singapore, Level 9, NUHS Tower Block, 1E Kent Ridge Road, Singapore, 119228 Singapore; 4 G. S. Kostiuk Institute of Psychology, National Academy of Educational Sciences of Ukraine, Kyiv, Ukraine; 5 Faculty of Social Sciences, Institute of Psychology, University of Silesia in Katowice, Katowice, Poland; 6 Braxia Scientific Corp, University of Toronto, University Health Network, Toronto, ON Canada; 7 Institute for Health Innovation and Technology (iHealthtech), National University of Singapore, Singapore, Singapore; 8 Mood Disorders Psychopharmacology Unit, University Health Network, Toronto, Canada; 9 University of Toronto, Toronto, Canada; 10 Brain and Cognition Discovery Foundation, Toronto, Canada; 11 Department of Healthcare Administration, Asia University, Taichung, Taiwan; 12 Department of Psychology, Asia University, Taichung, Taiwan

**Keywords:** Psychology, Health care

## Abstract

Ukraine has been embroiled in an increasing war since February 2022. In addition to Ukrainians, the Russo-Ukraine war has affected Poles due to the refugee crisis and the Taiwanese, who are facing a potential crisis with China. We examined the mental health status and associated factors in Ukraine, Poland, and Taiwan. The data will be used for future reference as the war is still ongoing. From March 8 to April 26, 2022, we conducted an online survey using snowball sampling techniques in Ukraine, Poland, and Taiwan. Depression, anxiety, and stress were measured using the Depression, Anxiety, and Stress (DASS)-21 item scale; post-traumatic stress symptoms by the Impact of Event Scale-Revised (IES-R) and coping strategies by the Coping Orientation to Problems Experienced Inventory (Brief-COPE). We used multivariate linear regression to identify factors significantly associated with DASS-21 and IES-R scores. There were 1626 participants (Poland: 1053; Ukraine: 385; Taiwan: 188) in this study. Ukrainian participants reported significantly higher DASS-21 (*p* < 0.001) and IES-R (*p* < 0.01) scores than Poles and Taiwanese. Although Taiwanese participants were not directly involved in the war, their mean IES-R scores (40.37 ± 16.86) were only slightly lower than Ukrainian participants (41.36 ± 14.94). Taiwanese reported significantly higher avoidance scores (1.60 ± 0.47) than the Polish (0.87 ± 0.53) and Ukrainian (0.91 ± 0.5) participants (*p* < 0.001). More than half of the Taiwanese (54.3%) and Polish (80.3%) participants were distressed by the war scenes in the media. More than half (52.5%) of the Ukrainian participants would not seek psychological help despite a significantly higher prevalence of psychological distress. Multivariate linear regression analyses found that female gender, Ukrainian and Polish citizenship, household size, self-rating health status, past psychiatric history, and avoidance coping were significantly associated with higher DASS-21 and IES-R scores after adjustment of other variables (*p* < 0.05). We have identified mental health sequelae in Ukrainian, Poles, and Taiwanese with the ongoing Russo-Ukraine war. Risk factors associated with developing depression, anxiety, stress, and post-traumatic stress symptoms include female gender, self-rating health status, past psychiatric history, and avoidance coping. Early resolution of the conflict, online mental health interventions, delivery of psychotropic medications, and distraction techniques may help to improve the mental health of people who stay inside and outside Ukraine.

## Introduction

**Figure 1:**
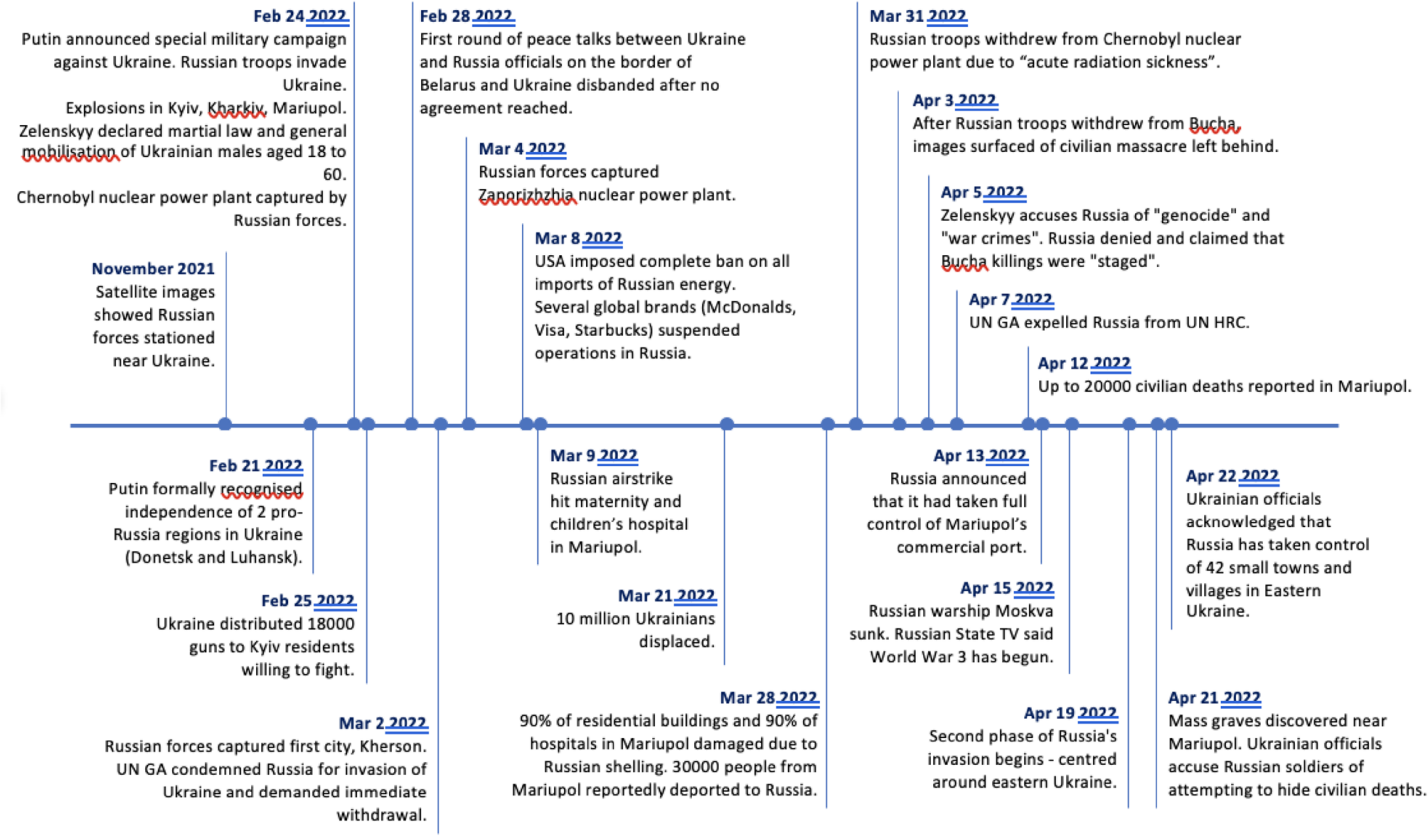
Chronology of events before and during Russia-Ukraine war in 2021 and 2022. *UN GA* United Nations General Assembly, *UN HRC* United Nations Human Rights Council.

After the independence of Ukraine in 1991, Russia and Ukraine conflicted with the Ukrainian Maidan Revolution in 2013, followed by the Russian annexation of Crimea and the occupation of the Luhansk and Donetsk regions of Ukraine in 2014. There have been ongoing tensions due to Russia objecting to Ukraine joining the North Atlantic Treaty Organization (NATO). This tension culminated in Russia launching a full-scale invasion of Ukraine on February 24, 2022^1^. At the time of recruitment for this study, the ongoing war was entering its second month and the events were summarized in Fig. 1. The lives of Ukrainians changed overnight—thousands volunteered to enlist in the armed forces, and millions had to flee from their homes as war broke out in their cities^2^. Missiles and explosives have been used in densely populated areas, leading to civilian deaths and injuries on top of military casualties^3^. Jain et al.^4^ pointed out that the number of civilian causalities outnumbered previous wars in Iraq, Iran, Afghanistan, and Vietnam^4^. The Russo-Ukrainian war has also given rise to the largest refugee crisis in Europe since World War II^5^. Of the 5 million who have fled to neighboring countries in the two months since the start of the war, more than half were taken in by Poland^6^. Besides physical health issues, many of these refugees require support for mental health problems due to trauma^7^.

The basis to conduct a study of the psychological impact of the war is based on the following theories. First, war-related trauma is a traumatic event that poses a threat to life or health^8^, by directly exposing an individual to violence^9^ and witnessing brutality. Direct exposure to war is a detrimental life event that can lead to long-term changes in mental well-being^10^, psychological damage^11^, and mental health disorders such as post-traumatic stress, depression, anxiety, and distress in adults and children^9,12^. Underlying factors within explanatory models of stress during wars include both direct trauma exposure and other psychosocial stressors such as financial loss. Second, emotional suffering related to war may occur not only due to direct exposure but also through indirect sources such as viewing war scenes via television or social media. Based on the indirect exposure theory, people who are concerned about war but live outside the war zone can develop adverse mental health consequences. Third, changes in the structure of society during the war often led to a breakdown of the existing protective networks, leading to depression and anxiety. Furthermore, the sense of identity of an individual has been stripped without any ability to prepare for it. This can also cause depression and anxiety. Fourth, individuals may overcome existing stress by developing coping mechanisms when facing war. According to Lazarus and Folkman’s (1984) theory of stress, an individual’s psychosocial reactions toward the war is due to the subjective evaluation of the meaning of the war, and therefore depend on the perceived ability to cope. Previous research found that the intensity of traumatic life events during the war and negative coping strategies yielded significant associations with post-traumatic stress^10^, leading to higher levels of psychiatric comorbidity and increased suicidality^13^. In contrast, coping strategies such as religious praying, and seeking the support of family members improve the mental health of people who witness war. Fifth, Hobfoll’s (1989) conservation of resources theory states that people differ not only in the strength of their stress reactions but also in the level of personal resources, which play an extremely important role in adaptation processes. The resources possessed by an individual, as well as their deficits, can have a significant impact on coping with war-induced stress and be influenced by cultural values. Psychological interventions should incorporate the cultural capacity to provide appropriate and effective mental health services to future victims of war. This theory supports a cross-cultural study to compare the psychological reactions of people from different countries towards the Russo-Ukrainian war.

A recent meta-analysis highlighted that mental health crisis during the wars is a public health issue. This meta-analysis found that more avoidance coping strategies were associated with increased depression among war verterans^13^. Bardi et al.^14^ found that cultural values predicted coping^15^ and it is important to conduct cross-cultural research to assess the impact of the Russo-Ukrainian war. Adverse long-term effects of war, besides psychiatric disorders, include the impairment of social functioning, poverty, malnutrition, health problems, and reduced quality of life^15^. Research findings suggest that refugees^11^ have a higher rate of depression, anxiety, and post-traumatic stress than the general population not affected by war^16^, due to traumatic war-related events and adverse post-war living conditions^12^, with probable post-traumatic stress persistence over time^17,18^. Civilians exposed to various wars are also at higher lifetime risk of psychiatric morbidity^19^. Based on the theoretical models and previous research findings, there are research questions related to the Russo-Ukrainian war. First, what factors are associated with the development of post-traumatic stress, depression, anxiety, and stress, differentiated by their proximity to a war zone? Second, what are the coping mechanisms adopted by different cultures?

First responders to war and combat veterans are at high risk of psychiatric morbidity due to increased exposure to distressing scenarios. Ukrainians have witnessed death, experienced separation from loved ones, and lost access to necessities^20^. Outside Ukraine, graphic images of the war on social media can also negatively impact the psychological health of people^21^. The fear and uncertainty created by the war are likely to have a lasting impact on the mental health of Ukrainians and people from other parts of the world, both actively being affected by the conflict and watching from afar. Additionally, the war in Ukraine is the first war in history to be reported almost continuously in the media, and the drastic scenes and images can be seen by virtually anyone with access to the Internet and television.

The psychological effects of the war can be felt by residents of other countries, although not on the same scale as by citizens of Ukraine, for obvious reasons. The closer their country of residence is to the site of the armed conflict, the more likely it is that people may be negatively affected by media coverage^22^. Poles may feel anxious about the war because of historical circumstances and because Poland received the largest number of migrating refugees from Ukraine since World War II. Most refugees were children and women, posing challenges to Poland’s society and the healthcare system^23^. In East Asia, the Taiwanese were watching the situation in Ukraine and worried that they were not prepared to face the potential invasion by the People’s Liberation Army from China, which is far larger and better equipped than the Taiwanese army^24^. Recently, the Chinese Defence minister informed their U.S. counterparts that China might start a war if Taiwan declares independence^25^. Taiwanese are in a peculiar geopolitical situation similar to that of Ukrainians and Poles. They are aware of the potential threat of war with China, just like Ukrainians and Poles facing Russian aggression. Military exercises conducted by China in the airspace and waters surrounding Taiwan in the last few months could be perceived as threatening by the country’s residents^26^. Moreover, the Taiwanese are emotionally linked to the Russo-Ukrainian war because a Taiwanese man who volunteered to fight in Ukraine died on the battlefield recently^27^. Therefore, the choice to investigate the mental health status among residents of the three countries was based on their similarities regarding the perceived threat of aggression from a neighbouring country, fears of a possible invasion, and the different geographic distances from Ukraine, where the current war is taking place.

The inclusion of representatives of different cultures is an additional asset of this research, as it allows more profound insight into possible similarities and differences in reactions to war. It is important to emphasize the ambiguity of the conclusions in the literature regarding possible cultural differences in response to the stress and trauma of war between representatives of different nations and a small number of studies devoted to it. On the one hand, previous studies emphasize that culture, media messages, cultural values, and the way society is organized affect people’s resilience to stress, stress response, and the coping process. Such effects are possible by influencing individuals’ identification of stressors, the assessment of the stressfulness of events, the types of resources from which to draw, and the individual response to stress^28,29^. Furthermore, a ccording to Weems et al.^30^, the context in which individuals experience a disaster can be an important factor influencing their responses to it^30^. Cultural context-dependent factors stemming from cultural norms and internalized values may influence certain responses and strategies to cope with trauma and stress during the Russian-Ukrainian war. On the other hand, according to Miller et al.^31^, there is no doubt that PTSD symptoms, especially symptoms involving intrusive re-experiencing of traumatic events (e.g. flashbacks, nightmares, recurring intrusive images or arousal, increased reactivity to stimuli, and sleep disturbances), are experienced by people facing trauma regardless of cultural context^31^.

In this study, we assessed and compared mental health status (e.g. depression, anxiety, stress, and post-traumatic stress), coping strategies, and views on the Russo-Ukrainian war in populations from Poland, Ukraine, and Taiwan after the outbreak of the war. We also identified demographic socio-and economic factors associated with depression, anxiety, stress, and post-traumatic stress levels in all study participants.

## Methods

This study follows the STrengthening the Reporting of OBservational studies in Epidemiology (STROBE) and the checklist can be found in the supplementary file.

### Study design and population

The study was conducted in three countries (Ukraine, Poland, and Taiwan) from March 8, 2022, to 26, April 2022. The surveys were conducted over a few days to ensure maximum participation. We recruited participants from the general population living in Ukraine, Poland, and Taiwan after the outbreak of the Russo-Ukrainian war.

### Procedure

In light of the ongoing conflict in Ukraine and the COVID-19 pandemic, potential respondents were electronically invited. Information about this study and survey was posted on social media (e.g. Facebook, LinkedIn, Twitter, Telegram, and Viber). Based on the snowball sampling strategy, participants were also encouraged to invite new respondents from their personal contacts and networks. The survey was conducted via two online survey platforms (i.e. Google Forms Online Survey on social media and the SWPS University of Social Sciences and Humanities SONA platform). The Institutional Review Board of SWPS University, Poland, provided the ethics approval for this research study (WKEB76/03/2022). Informed consent was obtained from all participants, and the data collected were anonymized and kept confidential. No incentives were offered to participants.

### Outcomes

This study used the 2022 Russo-Ukrainian war questionnaire developed by study team members in Ukraine, Poland, and Taiwan with external consultation with mental health experts in Canada and Singapore. The questionnaire consisted of questions related to (1) demographic data; (2) direct impact of the 2022 Russo-Ukrainian war; (3) the psychological impact of the Russo-Ukrainian war; (4) Help-seeking behavior during the war. The Impact of Event Scale-Revised (IES-R) was used to measure the psychological impact of the Russo-Ukrainian war, and this scale was previously validated in Polish^32^ and Taiwanese^33^. Our Cronbach α values for the IES-R intrusion, avoidance, and hyperarousal subscales were 0.88, 0.81 and 0.85, respectively. The Depression, Anxiety, and Stress Scale (DASS-21) was used to measure the participants’ mental health status. The DASS-21 was previously validated in Polish^32^ and Taiwanese^34^. The Coping Orientation to Problems Experienced Inventory (Brief-COPE) was used to look at the coping strategies in response to the Russo-Ukrainian war. The measures were distributed in Ukrainian, Polish, and Taiwanese language versions. Our Cronbach α values for the DASS-21 depression, anxiety, and stress subscales were 0.84, 0.90 and 0.80, respectively.

### Statistical analysis

For analysis of the differences in IES-R, DASS-21, and Brief-COPE scores, the one-way analysis of variance (ANOVA) was used to compare the mean scores between Ukrainian, Polish, and Taiwanese participants. Bonferroni correction was used when comparing all participants’ IES-R, DASS-21, and Brief-COPE scores. Categorical variables were presented as the percentage of responses to the questions, calculated based on the number of respondents per response to the number of total responses to the question. The Chi-square test was used for the comparison of the categorical variables. Multivariate linear regression was used to calculate the associations between IES-R and DASS scores with the demographic data and Brief-COPE scores in the three populations. Nationality was dummy coded, with Taiwanese set as reference, before entered last into the linear regression. Zero-order and partial correlation were also calculated from the multivariate regression. All tests were two-tailed, and a significance level of *p* < 0.05 was used. The statistical analysis was done on SPSS Statistic 28.0.

### Ethical approval

The study was conducted in accordance with the Declaration of Helsinki and approved by the Institutional Review Board of SWPS University, Poland (WKEB76/03/2022).

### Informed consent

Informed consent was obtained from all subjects involved in the study. Subjects are all above the age of 18 years, so no informed consent was needed from parents or guardians.

## Results

### Comparison of demographic data and psychosocial profiles

There were 1626 participants (Poland: 1053; Ukraine: 385; Taiwan: 188) in this study. The demographic data of study participants are summarized in Supplementary Table 1. The majority of participants were women (Overall: 74.95%; Poland: 76.1%; Ukraine: 81.6%; Taiwan: 55.3%); had a university education (Overall: 61.25%; Poland: 48.5%; Ukraine: 80.3%; Taiwan: 94.2%); without chronic diseases (Overall: 98.65%; Poland: 80.6%; Ukraine: 73.8%; Taiwan: 91%); without a past history of psychiatric illnesses (Overall: 60.82%; Poland: 50.8%; Ukraine: 73%; Taiwan: 92%); without exposure to a serious accident, life-threatening condition or disaster before the war (Overall: 75.79%; Poland: 73.2%; Ukraine: 76.4%; Taiwan: 88.8%). In Ukraine, the largest proportion of participants were without medical insurance (76.4%) and suffered from COVID-19 infection in the past 2 years (67%) (Table 1).

**Table 1:** Comparison of demographic data and psychosocial profile among Polish, Ukrainian, and Taiwanese participants (N = 1626).

Demographic data	Mean ± SD (Number (%))			*p*-value
	Poland (N = 1053)	Ukraine (N = 385)	Taiwan (N = 188)	
Gender				
Male	252 (23.9)	71 (18.4)	84 (44.7)	< 0.001
Female	801 (76.1)	313 (81.6)	104 (55.3)	
Age				
12–21 years	295 (28)	83 (21.6)	29 (15.4)	< 0.001
22–30 years	451 (42.8)	81 (21)	45 (23.9)	
31–40 years	157 (14.9)	78 (20.3)	69 (36.7)	
41–49 years	111 (10.5)	66 (17.1)	32 (17)	
50–59 years	32 (3)	33 (8.6)	9 (4.8)	
Above 60 years	7 (0.7)	44 (11.4)	4 (2.1)	
Education attainment				
None	0 (0)	0 (0)	0 (0)	NA
Primary school	13 (1.2)	1 (0.3)	0 (0)	0.083
Secondary school (Grades 7–9)	14 (1.3)	3 (0.8)	0 (0)	0.215
Upper secondary school (Grades 10–12)	516 (49)	72 (18.7)	4 (2.1)	< 0.001
College	0 (0)	0 (0)	6 (3.2)	NA
University: Bachelor	206 (19.6)	140 (36.4)	99 (52.7)	< 0.001
University: Master or PhD	304 (28.9)	169 (43.9)	78 (41.5)	< 0.001
Do any of your family members reside in Ukraine?				
Yes	45 (4.3)	376 (97.7)	0 (0)	< 0.001
No	1008 (95.7)	9 (2.3)	188 (100)	
Marital status				
Single	334 (31.7)	99 (25.7)	88 (46.8)	< 0.001
Married	213 (20.2)	171 (44.4)	62 (33)	< 0.001
In a relationship with a significant other	461 (43.8)	78 (20.3)	34 (18.1)	< 0.001
Divorced/separated	41 (3.9)	30 (7.8)	4 (2.1)	0.002
Widowed	4 (0.4)	7 (1.8)	0 (0)	0.006
Employment status				
Student	496 (47.1)	115 (29.9)	52 (27.7)	< 0.001
Employed	517 (49.1)	213 (55.3)	124 (66)	< 0.001
Unemployed	9 (0.9)	20 (5.2)	5 (2.7)	< 0.001
Homemaker	22 (2.1)	0 (0)	2 (1.1)	0.013
Farmers	2 (0.2)	21 (5.5)	0 (0)	< 0.001
Retired	7 (0.7)	16 (4.2)	5 (2.7)	< 0.001
Are you a refugee?				
Yes	13 (1.2)	97 (25.2)	1 (0.5)	< 0.001
No	1040 (98.8)	288 (74.8)	187 (99.5)	
Parental status				
No children	827 (78.5)	196 (50.9)	141 (75)	< 0.001
I have a child younger/older than 16 years	190 (18)	156 (40.5)	41 (21.8)	< 0.001
I have children younger and older than 16 years	36 (3.5)	33 (8.6)	6 (3.2)	< 0.001
Household size				
1 person	190 (18)	47 (12.2)	15 (8)	< 0.001
2 people	347 (33)	85 (22.1)	29 (15.4)	< 0.001
3–5 people	490 (46.5)	237 (61.6)	127 (67.6)	< 0.001
6 people or more	26 (2.5)	16 (4.2)	17 (9)	< 0.001
Family monthly income (USD)	2358.24 ± 8780.92	2939.05 ± 33,754.02	4907.77 ± 8932.27	0.203
Are you religious?				
Yes	339 (32.2)	217 (56.4)	80 (42.6)	< 0.001
No	714 (67.8)	168 (43.6)	108 (57.4)	
Please self-rate your current health status				
Very good	248 (23.6)	38 (9.9)	42 (22.3)	< 0.001
Good	382 (36.3)	110 (28.6)	78 (41.5)	0.004
Fair	210 (19.9)	151 (39.2)	61 (32.4)	< 0.001
Poor	181 (17.2)	73 (19)	6 (3.2)	< 0.001
Very poor	32 (3)	13 (3.4)	1 (0.5)	0.123
Do you have medical insurance?				
Yes	982 (93.3)	91 (23.6)	186 (98.6)	< 0.001
No	71 (6.7)	294 (76.4)	2 (1.1)	
Do you suffer from a chronic illness (e.g. heart disease, cancer etc.)?				
Yes	204 (19.4)	101 (26.2)	17 (9)	< 0.001
No	849 (80.6)	284 (73.8)	171 (91)	
Did you suffer from COVID-19 in the past 2 years?				
Yes	614 (58.3)	258 (67)	1 (0.5)	< 0.001
No	439 (41.7)	127 (33)	187 (99.5)	
Did you suffer from psychiatric illness in the past (e.g. depression, anxiety disorder, alcoholism, post-traumatic stress disorder etc.)?				
Yes	518 (49.2)	104 (27)	15 (8)	< 0.001
No	535 (50.8)	281 (73)	173 (92)	
Have you experienced a serious accident, life-threatening situation or disaster before?				
Yes	282 (26.8)	91 (23.6)	21 (11.2)	< 0.001
No	771 (73.2)	294 (76.4)	167 (88.8)	

*SD* standard deviation, *NA* not applicable, *USD* United States dollar.

### Comparison of levels of depression, anxiety, stress, post-traumatic stress, and coping

**Figure 2:**
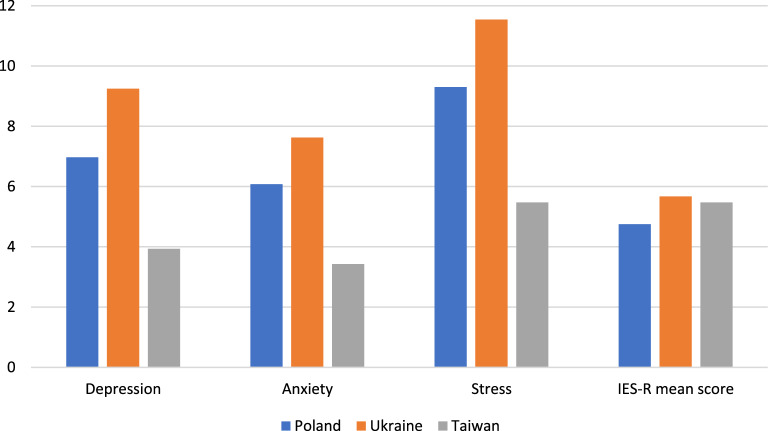
Summary of Depression, Anxiety, Stress, and mean IES-R scores between the three countries.

Table 2 compares the DASS-21, IE-R, and Brief COPE scores among Ukrainians, Poles, and Taiwanese. Ukrainians reported significantly higher DASS-21 total scores and DASS-21 anxiety, depression, and stress scores (*p* < 0.001) (Fig. 2). A significantly higher proportion of Ukrainian participants were classified as having depression (46.5%), anxiety (46.3%), and stress (28.6%) as compared to Taiwanese and Polish participants. However, Ukrainian participants reported significantly higher IES-R scores (41.36 ± 16.86) (*p* < 0.01), and the IES-R score of Taiwanese participants was slightly lower than Ukrainian participants (40.37 ± 16.86) even though Taiwan was not directly involved in the war. Around 57.2% of Polish, 73.2% of Ukrainian, and 56.9% of Taiwanese participants met the cut-off for post-traumatic stress. Surprisingly, Taiwanese participants reported significantly higher avoidance scores than Polish and Ukrainian participants (*p* < 0.001). Nevertheless, a significantly higher member of Ukrainian participants (73.2%) met the diagnostic criteria for post-traumatic stress disorder (*p* < 0.001). Taiwanese participants reported significantly higher scores in problem-focused, emotion-focusing, and avoidant coping (*p* < 0.001).

**Table 2:** Comparison of DASS-21, IES-R and Brief-COPE scores among Polish, Ukrainian, and Taiwanese participants (N = 1626).

Psychosocial profile	Mean ± SD (Number (%))			*p*-value
	Poland (N = 1053)	Ukraine (N = 385)	Taiwan (N = 188)	
DASS-21 score	44.71 ± 27.48	56.84 ± 25.14	25.66 ± 24.21	< 0.001
Depression	6.97 ± 5.06	9.25 ± 4.67	3.93 ± 4.41	< 0.001
Anxiety	6.08 ± 4.89	7.63 ± 4.85	3.43 ± 3.78	< 0.001
Stress	9.30 ± 5.22	11.54 ± 4.59	5.47 ± 4.74	< 0.001
Depression				
> 9	305 (29)	179 (46.5)	21 (11.2)	< 0.001
δ 9	748 (71)	206 (53.5)	167 (88.8)	< 0.001
Anxiety				
> 7	384 (36.5)	186 (46.3)	28 (14.9)	< 0.001
δ 7	669 (63.5)	199 (51.7)	160 (85.1)	< 0.001
Stress				
> 14	183 (17.4)	110 (28.6)	7 (3.7)	< 0.001
δ 14	870 (82.6)	275 (71.4)	181 (96.3)	< 0.001
IES-R score	35.01 ± 15.15	41.36 ± 14.94	40.37 ± 16.86	< 0.001
Mean IES-R score	4.75 ± 2.09	5.67 ± 2.07	5.47 ± 2.30	< 0.001
Avoidance	1.65 ± 0.76	1.62 ± 0.74	1.89 ± 0.81	< 0.001
Intrusion	1.60 ± 0.86	2.05 ± 0.89	1.87 ± 0.85	< 0.001
Hyperarousal	1.50 ± 0.87	2.00 ± 0.89	1.70 ± 0.81	< 0.001
IES-R score				
> 32 (Cut-off for post-traumatic stress)	602 (57.2)	282 (73.2)	107 (56.9)	< 0.001
δ 32	451 (42.8)	103 (26.8)	81 (43.1)	< 0.001
Brief-COPE score	34.36 ± 12.08	38.69 ± 11.33	63.49 ± 15.13	< 0.001
Mean brief-COPE score	3.64 ± 1.31	4.13 ± 1.22	6.75 ± 1.60	< 0.001
Problem-focused coping	1.47 ± 0.65	1.80 ± 0.61	2.79 ± 0.79	< 0.001
Emotion-focused coping	1.30 ± 0.46	1.42 ± 0.45	2.36 ± 0.62	< 0.001
Avoidant coping	0.87 ± 0.53	0.91 ± 0.50	1.60 ± 0.47	< 0.001

*DASS-21* depression, anxiety, stress scale, *IES-R* impact of event scale-revised, *Brief-COPE* brief coping orientation to problems experienced questionnaire.

### Comparison of impact, help-seeking behavior, and personal views on the war

Table 3 compares the impact of and views on the Russo-Ukrainian war among participants from Poland, Ukraine, and Taiwan. Regarding the impact of the war, for participants from Ukraine, around 48.3% knew a person who was killed; 91.2% knew a person who was endangered; 81% reported a negative impact on their income, and 72% attributed the war as a major cause of distress. Regarding the psychological impact of the war, although Taiwanese and Polish participants were not directly affected by the war, 80.3% of Polish and 54.3% of Taiwanese participants were distressed by media war scenes. Around 89.4% of Taiwanese, 82.6% of Polish, and 69.9% of Ukrainian participants agreed that media exposure to the current war caused psychological trauma. A significantly higher proportion of Ukrainian participants reported the following psychological impact (*p* < 0.001): 88.3% of Ukrainian participants were distressed by the war noises; 36% reported poor sleep quality, and 50.1% felt angry with the war as compared to Poles and Taiwanese. Although 59.2% of Ukrainian participants developed a mental health problem due to the war, 52.5% of Ukrainian participants would not seek professional help.

**Table 3:** Comparison of direct impact, psychological impact, help seeking behaviour and personal views on the war among Polish, Ukrainian and Taiwanese participants (n = 1626).

Demographic data	Number (%)			*p*-value
	Poland (N = 1053)	Ukraine (n = 385)	Taiwan (n = 188)	
I. Direct impact of the 2022 Russia-Ukraine War				
Do you know a person/friend/relative who was killed in the current Ukraine crisis?				
Yes	35 (3.3)	186 (48.3)	1 (0.5)	< 0.001
No	1018 (96.7)	199 (51.7)	187 (99.5)	
Do you know a person/friend/relative who was injured in the current war in Ukraine?				
Yes	47 (4.5)	181 (47)	2 (1.1)	< 0.001
No	1006 (95.5)	204 (53)	186 (98.6)	
Do you know a person/friend/relative who is being endangered in the current war in Ukraine?				
Yes	343 (32.6)	351 (91.2)	4 (2.1)	< 0.001
No	710 (67.4)	34 (8.8)	184 (97.9)	
Is your life endangered in the current war in Ukraine?				
Yes	37 (3.5)	223 (57.9)	2 (1.1)	< 0.001
No	1016 (96.5)	162 (42.1)	186 (98.6)	
Were you forced to separate from your family or loved ones during the current war in Ukraine?				
Yes	11 (1)	237 (61.6)	2 (1.1)	< 0.001
No	1042 (99)	148 (38.4)	186 (98.6)	
Does the war in Ukraine have a negative impact on your income?				
Yes	299 (28.4)	312 (81)	23 (12.2)	< 0.001
No	754 (71.6)	73 (19)	165 (87.8)	
Does the war in Ukraine affected your financial security?				
Yes	722 (68.6)	356 (92.5)	27 (14.4)	< 0.001
No	331 (31.4)	29 (7.5)	161 (85.6)	
Are you distressed by the war noises in Ukraine?				
Yes	721 (68.5)	340 (88.3)	89 (47.3)	< 0.001
No	332 (31.5)	45 (11.7)	99 (52.7)	
II. Psychological impact of the 2022 Russia-Ukraine War				
Are you distressed by the media war scenes from Ukraine?				
Yes	846 (80.3)	321 (83.4)	102 (54.3)	< 0.001
No	207 (19.7)	64 (16.6)	86 (45.7)	
Are you distressed when you hear the news or see people who were killed during the Ukraine crisis?				
Yes	870 (82.6)	358 (93)	168 (89.4)	< 0.001
No	183 (17.4)	27 (7)	20 (10.6)	
Do you consider the war in Ukraine to be a major cause of your distress lately?				
No	241 (22.9)	15 (3.9)	133 (70.7)	< 0.001
Partial	592 (56.2)	91 (23.6)	53 (28.2)	< 0.001
Yes, totally	220 (20.9)	279 (72.5)	2 (1.1)	< 0.001
Is the media exposure to the current war in Ukraine psychological traumatic?				
Agree	442 (42)	269 (69.9)	46 (24.5)	< 0.001
Neutral of no comment	293 (27.8)	76 (19.7)	84 (44.7)	< 0.001
Disagree	318 (30.2)	40 (10.4)	58 (30.9)	< 0.001
Your sleep quality since the war in Ukraine started is:				
Good	285 (27.1)	41 (10.6)	44 (23.4)	< 0.001
Average	625 (59.4)	205 (53.2)	136 (72.3)	< 0.001
Poor	143 (13.6)	139 (36.1)	8 (4.3)	< 0.001
Rumination about the current war in Ukraine				
I feel angry about the current war in Ukraine				
Nearly all the time	361 (34.3)	193 (50.1)	17 (9)	< 0.001
Sometimes	542 (51.5)	154 (40)	90 (47.9)	< 0.001
Rarely	101 (9.6)	26 (6.8)	62 (33)	< 0.001
Not at all	49 (4.7)	12 (3.1)	19 (10.1)	0.001
I feel injustice about the current war in Ukraine				
Nearly all the time	788 (74.8)	339 (88.1)	40 (21.3)	< 0.001
Sometimes	194 (18.4)	29 (7.5)	101 (53.7)	< 0.001
Rarely	38 (3.6)	9 (2.3)	35 (18.6)	< 0.001
Not at all	33 (3.1)	8 (2.1)	12 (6.4)	0.023
Help seeking behavior during the war in Ukraine				
Have you developed a mental health problem (e.g. depression, anxiety, post-traumatic stress) due to the war in Ukraine?				
Yes	216 (20.5)	228 (59.2)	7 (3.7)	< 0.001
No	837 (79.5)	157 (40.8)	181 (96.3)	
Would you seek professional help for your mental health problem related to the war in Ukraine?				
Yes	600 (57)	183 (47.5)	8 (4.3)	< 0.001
No	453 (43)	202 (52.5)	180 (95.7)	
If you want to seek professional help for your mental problems, which mental healthcare professionals would you consult?				
Psychiatrists	466 (25.5)	70 (13.8)	57 (30.3)	< 0.001
Clinical psychologists or counselors	810 (44.2)	197 (38.9)	92 (48.9)	< 0.001
General practitioners or family doctors	81 (4.4)	17 (3.4)	10 (5.3)	0.065
Social workers	12 (0.7)	16 (3.2)	5 (2.7)	0.001
Nurses	6 (0.3)	4 (0.8)	2 (1)	0.561
Religious leaders	59 (3.2)	46 (9.1)	5 (2.7)	< 0.001
Online psychotherapy	388 (21.2)	143 (28)	11 (5.9)	< 0.001
Others	9 (0.5)	14 (2.8)	6 (3.2)	< 0.001
III. Personal views towards the Russia-Ukraine war				
Do you fear that war may break out in your country/region soon?				
Yes	682 (64.8)	333 (86.5)	116 (61.7)	< 0.001
No	371 (35.2)	52 (13.5)	72 (38.3)	
You predict the war in Ukraine crisis will end in:				
A few days	10 (0.9)	3 (0.8)	3 (1.6)	0.637
One to two weeks	41 (3.9)	16 (4.2)	11 (5.9)	0.466
1 month	172 (16.3)	95 (24.7)	49 (26.1)	< 0.001
6 months	207 (19.7)	80 (20.8)	50 (26.6)	0.097
1 year	57 (5.4)	22 (5.7)	17 (9)	0.148
2 years	37 (3.5)	3 (0.8)	3 (1.6)	0.011
More than 2 years	72 (6.8)	11 (2.9)	10 (5.3)	0.015
I do not know	457 (43.4)	155 (40.3)	45 (23.9)	< 0.001
Do you think that the current war in Ukraine may lead to the outbreak of World War III?				
Likely	382 (36.3)	261 (67.8)	75 (39.9)	< 0.001
Neutral or no comment	366 (34.8)	50 (13)	49 (26.1)	< 0.001
Unlikely	305 (29)	74 (19.2)	64 (34)	< 0.001
Do you think that the current war in Ukraine may lead to the use of nuclear weapons?				
Likely	273 (25.9)	151 (39.2)	81 (43.1)	< 0.001
Neutral or no comment	345 (32.8)	105 (27.3)	46 (24.5)	0.022
Unlikely	435 (41.3)	129 (33.5)	61 (32.4)	0.005
Average time spent on news related to the war in Ukraine crisis per day				
0 h per day	94 (8.9)	10 (2.6)	57 (30.3)	< 0.001
Up to 1 h	556 (52.8)	75 (19.5)	99 (52.7)	< 0.001
1–2 h per day	256 (24.3)	126 (32.7)	23 (12.2)	< 0.001
3–5 h per day	109 (10.4)	114 (29.6)	8 (4.3)	< 0.001
6–8 h per day	26 (2.5)	32 (8.3)	1 (0.5)	< 0.001
8–10 h per day	6 (0.6)	7 (1.8)	0 (0)	0.027
Above 10 h per day	6 (0.6)	21 (5.5)	0 (0)	< 0.001
What is your most preferred source of information about the war in Ukraine?				
Social media	622 (25.1)	252 (31.7)	59 (31.4)	< 0.001
Internet	857 (34.6)	268 (33.7)	89 (47.3)	< 0.001
Television	359 (14.5)	107 (13.5)	34 (18.1)	< 0.001
Radio	177 (7.2)	26 (3.3)	2 (1.1)	< 0.001
Newspaper	70 (2.8)	6 (0.8)	1 (0.5)	< 0.001
Friends	311 (12.6)	103 (13)	2 (1.1)	< 0.001
Others	78 (3.2)	33 (4)	1 (0.5)	< 0.001

Regarding the views on the war, 43.4% of Polish and 40.3% of Ukrainian participants did not know when the war would end. Around 67.8% of Ukrainian and 39.9% of Taiwanese participants thought the current war would lead to the third world war. Significantly more Taiwanese participants (43.1%) thought that the current war would lead to the use of nuclear weapons (*p* < 0.001). Most Polish (52.8%) and Taiwanese (52.7%) participants spent up to 1 h per day, while 32.7% of Ukrainian participants spent up to 1–2 h on the news related to war. The Internet was rated as the most popular source of information about the war (Taiwanese: 47.3%, Polish: 34.6%, and Ukrainian: 33.7%).

### Linear regression analysis with total DASS-21 scores as the dependent variable

Table 4 shows the results of multivariate linear regression analysis using DASS-21 scores as the dependent variable. The multivariate linear regression analysis found that female gender, Ukrainian and Polish nationality, household size, self-rating health status, past psychiatric history, presence of chronic illness, emotion-focused coping, and avoidant coping was significantly associated with higher total DASS-21 scores after adjustment of other variables (*p* < 0.05). In contrast, age, presence of social support, and problem-focused coping were significantly associated with lower total DASS-21 scores after adjustment of other variables (*p* < 0.05).

**Table 4:** Multivariate regression analysis for DASS-21 scores by linear regression analysis (all groups).

	Multivariate regression (R^2^ = 0.463)			
	Slope (SE)	Zero-order correlation	Partial correlation	*p*-value
Gender (Female)	6.072 (1.265)	0.200	0.119	< 0.001**
Age	− 1.111 (0.698)	− 0.053	− 0.040	0.111
Education attainment	0.564 (0.505)	− 0.062	0.028	0.265
Nationality				
Polish vs. Taiwanese	27.541 (2.144)	-0.031	0.306	< 0.001**
Ukrainian vs. Taiwanese	36.055 (2.455)	0.231	0.344	< 0.001**
Marital status	− 0.510 (0.727)	0.019	0.019	0.483
Employment status	− 0.292 (0.716)	− 0.033	− 0.018	0.684
Refugee status	2.686 (2.285)	0.131	0.029	0.240
Parental status	2.351 (1.367)	0.027	0.043	0.086
Household size	0.911 (0.746)	0.004	0.030	0.041*
Family monthly income (USD)	− 0.000 (0.000)	− 0.005	− 0.015	0.558
Having a religious faith	− 0.517 (1.148)	0.051	− 0.011	0.653
Self-rating health status	3.888 (0.511)	0.283	0.187	< 0.001**
Presence of medical insurance	− 3.339(1.741)	− 0.149	− 0.048	0.055
Presence of chronic illness	3.744 (1.371)	0.139	0.068	0.011*
Past COVID-19 infection	1.522 (1.130)	0.109	0.034	0.178
Past psychiatric history	7.843 (1.171)	0.244	0.165	< 0.001**
Experience of past trauma	0.828 (1.255)	0.042	0.016	0.510
Presence of social support	− 9.339 (2.005)	− 0.108	− 0.116	< 0.001**
Brief-COPE score				
Problem-focused coping	− 4.559 (1.113)	0.005	− 0.102	< 0.001**
Emotion-focused coping	7.043 (1.639)	0.113	0.107	< 0.001**
Avoidant coping	24.123 (1.171)	0.395	0.458	< 0.001**

*p < 0.05.

**p < 0.01.

### Linear regression analysis with the IES-R scores as the dependent variable

Table 5 shows the multivariate linear regression analysis results using the IES-R as the dependent variable. The multivariate linear regression analysis found that female gender, Ukrainian and Polish nationalities, household size, self-rating health status, presence of medical insurance, past psychiatric history, problem-focused and avoidant coping were associated with higher IES-R scores after adjustment of other variables (*p* < 0.05).

**Table 5:** Multivariate regression analysis for IES-R scores by linear regression analysis (all groups).

	Multivariate regression (R^2^ = 0.397)			
	Slope (SE)	Zero-order correlation	Partial correlation	*p*-value
Gender (Female)	6.263 (0.736)	0.243	0.208	< 0.001**
Age	− 0.532 (0.406)	0.007	− 0.033	0.190
Education attainment	0.435 (2.94)	0.050	0.037	0.139
Nationality				
Polish vs. Taiwanese	4.776 (1.248)	− 0.184	0.095	< 0.001**
Ukrainian vs. Taiwanese	10.372 (1.429)	0.157	0.178	< 0.001**
Marital status	0.570 (0.337)	0.014	0.042	0.090
Employment status	− 0.259 (0.423)	− 0.008	− 0.015	0.541
Refugee status	0.716 (1.330)	0.100	0.013	0.590
Parental status	0.709 (0.797)	0.057	0.022	0.374
Household size	1.020 (0.434)	0.079	0.059	0.004**
Family monthly income (USD)	− 0.000 (0.000)	0.027	-0.009	0.713
Having a religious faith	− 0.585 (0.668)	0.062	− 0.022	0.381
Self-rating health status	1.477 (0.298)	0.187	0.123	< 0.001**
Presence of medical insurance	1.377 (1.013)	− 0.047	0.034	0.044*
Presence of chronic illness	− 0.205 (0.798)	0.033	− 0.006	0.797
Past COVID-19 infection	0.716 (0.657)	− 0.006	0.027	0.276
Past psychiatric history	1.530 (0.681)	0.075	0.056	0.025*
Experience of past trauma	− 0.013 (0.731)	− 0.025	− 0.001	0.986
Presence of social support	− 0.220 (1.167)	0.005	− 0.005	0.850
Brief-COPE score				
Problem-focused coping	1.035 (0.648)	0.271	0.040	< 0.001**
Emotion-focused coping	1.482 (0.954)	0.321	0.039	0.121
Avoidant coping	14.150 (0.682)	0.537	0.460	< 0.001**

*p < 0.05.

**p < 0.01.

## Discussion

To the best of our knowledge, this is the first study comparing depression, anxiety, stress, and post-traumatic stress levels among people from Poland, Ukraine, and Taiwan. The key findings of this study are summarized as follows: Firstly, as expected, Ukrainian participants reported significantly higher scores for depression, anxiety, stress, and post-traumatic stress. Second, although Taiwanese participants were not directly involved in the war, their mean IES-R scores were slightly lower than Ukrainian participants. Taiwanese reported significantly higher avoidance scores than the Polish and Ukrainian participants; Third, more than half of the Taiwanese and Polish participants were distressed by the media war scenes. Fourth, more than half of the Ukrainian participants would not seek psychological help despite a significantly higher prevalence of mental health sequelae. Fifth, female gender, Ukrainian and Polish nationality, household size, self-rating health status, past psychiatric history, and avoidance coping were significantly associated with both higher DASS-21 and IES-R scores after adjustment of other variables.

This study found that 46.5 and 46.3% of Ukrainian were classified as having a high score for depression and anxiety, respectively. A previous retrospective study found that civilians who lived in a war zone during WWII had significantly higher lifetime risks than other respondents with major depressive and anxiety disorders^19^. Although the aforementioned finding was expected as Ukrainians were directly affected by the war and faced the destruction of homes, hospitals, and livelihoods, it was surprising that Taiwanese participants were significantly associated with high IES-R scores and adopted the highest level of avoidant coping. The high IES-R score suggests high post-traumatic stress as the Taiwanese were watching the war in Ukraine with many concerns^35^ and one Taiwanese soldier died in the war. This finding is supported by previous studies, which reported that frequent exposure to graphic media images of violence and deaths after terrorist attacks and wars could result in psychological stress^36,37^. Taiwanese participants might see a parallel future with Ukrainian and try to avoid discussing potential war with China. The following observation justifies the avoidance coping strategy. The Global Taiwan Institute analyzed the current situation and concluded that Taiwan is uncertain how far Europe and the United States will go to aid Taiwan in its hour of need if a potential war breaks out, facing a strong Sino-Russian alliance^38^.

Besides the impact of graphics, the perceived level of controllability and culture might explain the choice of coping strategy. As avoidance coping is particularly associated with a low level of controllability of a situation^39^, it might be adopted by the Taiwanese participants during the time of uncertainty. The fact that the Taiwanese scored the highest on avoidance coping can be attributed to cultural differences compared to the Ukrainians and Poles. The previous study by Bardi and Guerra (2011) showed that cultural values are related to coping strategies^14^. Avoidance coping was more frequently used in non-Western cultural groups, where the value of embeddedness emphasizes tradition and group interests, while the social hierarchy in Asian culture encourages people to cope more passively.

This study found that more than half of the Ukrainian participants would not seek psychological help despite a significantly higher prevalence of depression, anxiety, stress, and post-traumatic stress. This finding raises concerns because Ukrainians would be unable to express and process their psychiatric symptoms during the constantly evolving Russo-Ukrainian war, which might lead to worsening psychiatric symptoms. Ukrainians might suppress their traumatic experiences due to shame and fear^40^ and a lack of awareness of psychiatric morbidity. When reviewing the state of trauma and trauma care in Ukraine, Schäfer et al.^41^ noted that the Ukrainian population might not recognize the problems of war-related psychological violence and believe that the end of the war would terminate the psychological impact without the need to seek professional help^41^. The above phenomenon suggests that a vicious cycle might exist in people who were affected by traumatic war-related events. The psychological distress may limit the ability of the people to adopt effective coping strategies, which can lead to further deterioration of mental and physical functioning. The regression analysis found that high self-rating health status (i.e. physical health was associated with higher DASS-21 and IES-R scores. This finding suggested that people might prioritize healthcare resources for physical injuries and diseases instead of mental health during the Russo-Ukraine war. Conventional mental health services include screening by primary care physicians, implementation of treatment guidelines, psychoeducation, community support, and psychiatric hospitalization for severe cases that are not applicable in conflicted areas. Therefore, the aforementioned interventions should be supplemented with psychological support which aimed at providing immediate relief to people affected by a military conflict^41^. It may not be possible to rebuild the mental health infrastructure in Ukraine when the war is still ongoing. Due to the Internet, mental health professionals from other countries, with the assistance of translators, can provide online psychological support in the format of crisis intervention as well as individual and group therapies. As social support is associated with fewer mental health sequelae, overseas Ukrainians can form an online support group for fellow citizens who still stay in Ukraine. The United Nations (U.N.), World Health Organization (WHO), governments from western countries, and humanitarian NGOs (e.g., Red Cross, Doctors without Borders) should provide online mental health support and deliver psychotropic medications (e.g. antidepressants) to Ukrainians. Furthermore, professionals specialized in trauma-focused therapies and trauma care for survivors should be involved in the process of providing psychosocial services, as traumatized migrants and refugees require long-term therapy to counteract the adverse war-induced consequences for the society at large^41^. For people who live outside the war zones, such as the Poles and Taiwanese, physical and social distraction^37^ would reduce the time spent on the news and graphic media images related to the Russo-Ukraine war. Our research findings support online anger management and digital cognitive behavior therapy (CBT)^42^ to treat anger and insomnia, two common psychological problems associated with the current war. Post-migration mental health services and infrastructures will be helpful to Ukrainian refugees who have fled to Poland. Based on our regression analysis, priorities for psychological and psychiatric interventions should be given to people with a past psychiatric and medical history as they are at risk for mental health sequelae. Online cognitive therapy should be offered to challenge and reduce avoidant coping.

The female gender was significantly associated with higher DASS-21 and IES-R scores after adjustment of other variables. Outside the war zones, the female gender was associated with a higher prevalence of depression in the community in 30 countries between 1994 and 2014^43^. In the war zone, women faced gender-specific risks as potential victims of rape, sexual abuse, targeted killing, widowhood of deceased soldiers, and pregnancy-related complications due to poor antenatal and postnatal healthcare^4^.

This study has several limitations. First, this study is cross-sectional, and the Russo-Ukrainian war had not ended at the time of writing. This study measured the immediate psychological impact, and further longitudinal study is required to monitor long-term psychiatric and psychological sequelae of Ukrainians and their association with post-war socio-economic status^44^. A previous retrospective study found that the association of war exposure with MDD was the strongest in the early years after WWII, whereas the association with anxiety disorders increased over time^19^. Second, the sample size is relatively smaller in Ukraine (n = 385) and Taiwan (n = 188). As this study was conducted during the outbreak of the Russo-Ukrainian war, snowball sampling was deemed the best and most appropriate technique to collect data. This sampling method might have created a bias in sample diversity and resulted in the under-representation of respondents from smaller or hard-to-reach networks^45^. Similar to challenges in estimating the number of civilian casualties in modern warfare^46^, it would be equally challenging to estimate the prevalence of psychiatric morbidity among the civilian population during the Russo-Ukraine war. Third, this study did not explore other psychiatric morbidities, including suicidal ideation or attempts, eating disorders, substance abuse, addiction, and gambling^4^. Fourth, this study focused on adult civilians due to consent and logistic requirements. We did not recruit children, minors, and the elderly. Further studies are required to assess the mental health of different age groups during the Russo-Ukraine war. Finally, due to a large number of study variables, some of the variables such as chronic medical diseases is a generic term and did not refer to specific disorders.

## Conclusion

In conclusion, our findings show major mental health sequelae in Ukrainian, Poles, and Taiwanese with the ongoing Russo-Ukraine war. Risk factors associated with developing depression, anxiety, stress, and post-traumatic stress symptoms include female gender, self-rating health status, past psychiatric history, and avoidance coping. This service gap will require a substantial increase in mental health service capacity, but this will be a huge challenge to Ukraine as the war is still ongoing. For countries outside Ukraine, mental health professionals should be vigilant in recognizing the mental health sequelae of refugees from Ukraine by offering initial mental health assessments, ensuring their safety, and restoring their daily routines. Early resolution of the conflict, online mental health interventions, delivery of psychotropic medications, distraction techniques, and adaptive coping strategies may help to improve the mental health of people who stay inside and outside Ukraine. The knowledge gap regarding the impact of the Russo-Ukraine war on young people and the elderly needs to be addressed in future studies. As there is no end date for the Russo-Ukraine war and the possibility of extension of the front line to other parts of the world, ongoing monitoring and surveillance of mental health sequelae is necessary. An in-depth understanding of the effect of war and its consequences, among other innumerable mental health problems, is necessary to develop consistent and effective coping strategies.

## Supplementary Information

Supplementary Table 1.

## Data Availability

The data presented in this study are available on request from the corresponding author.
