# Supplementary material for: Depression, anxiety and post-traumatic stress during the 2022 Russo-Ukrainian war, a comparison between populations in Poland, Ukraine, and Taiwan
Source: Sci Rep. 2023 Mar 3;13:3602. doi: 10.1038/s41598-023-28729-3 (PMC9982762; doi:10.1038/s41598-023-28729-3)
Supplement: Supplementary file 1 — Supplementary Table 1. [file 41598_2023_28729_MOESM1_ESM.pdf]

Supplementary Table 1. Comparison of demographic data, impact, rumination and help seeking behavior between Polish, Ukrainian and Taiwanese respondents

Demographic data	Mean±SD (Number (%))			
	Poland (n=1053)	Ukraine (n=385)	Taiwan (n=188)	P
**Nationality**				
Polish	1049 (99.6)	2 (0.5)	0 (0)	<0.001
Ukrainian	0 (0)	362 (94)	0 (0)	
Taiwanese	0 (0)	0 (0)	185 (98.4)	
Others	4 (0.4)	21 (5.5)	3 (1.6)	
**Current residential country**				
Poland	1033 (98.1)	45 (11.7)	0 (0)	<0.001
Ukraine	1 (0.1)	292 (75.8)	0 (0)	
Taiwan	0 (0)	0 (0)	186 (98.9)	
Others	19 (1.8)	48 (12.5)	2 (1.1)	
**Were you originated from Ukraine?**				
Yes	31 (2.9)	376 (97.7)	1 (0.5)	<0.001
No	1022 (97.1)	9 (2.3)	187 (99.5)	
**Was any of your family members originated from Ukraine?**				
Yes	78 (7.4)	379 (98.4)	1 (0.5)	<0.001
No	975 (92.6)	6 (1.6)	187 (99.5)	
**Was any of your friends originated from Ukraine?**				
Yes	389 (36.9)	381 (99)	7 (3.7)	<0.001
No	664 (63.1)	4 (1)	181 (96.3)	
**Do any of your friends reside in Ukraine?**				
Yes	162 (15.4)	382 (99.2)	6 (3.2)	<0.001
No	891 (84.6)	3 (0.8)	182 (96.8)	
**There is a person (s) next to me who gives me support**				
No, there is not such a person	73 (6.9)	41 (10.6)	13 (6.9)	0.059
Yes, there is such a person	980 (93.1)	344 (89.4)	175 (93.1)	0.059
**Impact of the 2022 War in Ukraine**				
**How many times have you had to relocate due to the current Ukraine crisis and war?**				
Never	1040 (98.9)	180 (46.8)	NA	<0.001
Once	9 (0.9)	112 (29.1)		<0.001
Twice	2 (0.1)	55 (14.3)		<0.001
Three times or more	2 (0.1)	38 (9.9)		<0.001
**The main cause of my stress at this moment is**				
Educational	28 (2.7)	9 (2.3)	38 (20.2)	<0.001
Economical	213 (20.2)	41 (10.6)	77 (41)	<0.001
Social	99 (9.4)	32 (8.3)	24 (12.8)	0.227

War-Related	515 (48.9)	269 (69.9)	25 (13.3)	<0.001
Medical	10 (0.9)	8 (2.1)	11 (5.9)	<0.001
Others	188 (17.9)	26 (6.8)	13 (6.9)	<0.001
**Can you deal with your emotions no matter how bad it gets in the war in Ukraine?**				
Yes	863 (82)	315 (81.8)	167 (88.8)	0.062
No	190 (18)	70 (18.2)	21 (11.2)	
**What is the attitude towards Russian invasion into Ukraine?**				
Support Russian invasion	5 (0.5)	0 (0)	1 (0.5)	0.390
Against Russian invasion	1021 (97)	381 (99)	160 (85.1)	<0.001
I do not have an opinion	27 (2.6)	4 (1)	27 (14.4)	<0.001
**The United Nation, NATO and European Union have done enough to support Ukraine**				
Agree	180 (17.1)	31 (8.1)	9 (4.8)	<0.001
Neutral or no comment	408 (38.7)	138 (35.8)	75 (39.9)	0.531
Disagree	465 (44.2)	216 (56.1)	104 (55.3)	<0.001
**Have you participated in protests against the Russian invasion?**				
Yes	176 (16.7)	115 (29.9)	11 (5.9)	<0.001
No	877 (83.3)	270 (70.1)	177 (94.1)	
**I feel insecure about the current war in Ukraine**				
Nearly all the time	158 (15)	142 (36.9)	12 (6.4)	<0.001
Sometimes	536 (50.9)	160 (41.6)	86 (45.7)	0.006
Rarely	237 (22.5)	50 (13)	57 (30.3)	<0.001
Not at all	122 (11.6)	33 (8.6)	33 (17.6)	0.007
**I feel hopeless about the current war in Ukraine**				
Nearly all the time	569 (54)	176 (45.7)	3 (1.6)	<0.001
Sometimes	360 (34.2)	121 (31.4)	54 (28.7)	0.265
Rarely	67 (6.4)	60 (15.6)	88 (46.8)	<0.001
Not at all	57 (5.4)	28 (7.3)	43 (22.9)	<0.001
**Help seeking behavior during the war in Ukraine**				
**If you do NOT want to seek professional help for mental problems, what is the reason?**				
I do not have a mental health problem	391 (37.1)	60 (15.6)	67 (35.6)	<0.001
I can handle it myself	230 (21.8)	149 (38.7)	55 (29.3)	<0.001
I prefer to seek help from friends or family members	90 (8.5)	27 (7)	34 (18.1)	<0.001
I do not have money to pay for mental health service	95 (9)	48 (12.5)	9 (4.8)	0.010
Long waiting time	29 (2.8)	1 (0.3)	1 (0.5)	0.003
No access to mental health service	5 (0.5)	5 (1.3)	0 (0)	0.108
Concerns about privacy issue	12 (1.1)	6 (1.6)	5 (2.7)	0.257
Mental health service is ineffective	22 (2.1)	8 (2.1)	3 (1.6)	0.904
	40 (3.8)	12 (3.1)	7 (3.7)	0.827

I am too busy and no time for mental health service	17 (1.6)	1 (0.3)	4 (2.1)	0.089
I do not have a health insurance that covers mental health service	7 (0.7)	46 (11.9)	0 (0)	<0.001
Mental health service is disrupted by the war	115 (10.9)	22 (5.7)	3 (1.6)	<0.001
Others				
